# The Mystery of a Negative Workup: A Diagnostic Dilemma in Seronegative Immune-Mediated Transverse Myelitis

**DOI:** 10.7759/cureus.103440

**Published:** 2026-02-11

**Authors:** Niyas Khalid Ottu Para, Uvashree Shrinivas, Fareeda Udyavar Karoda

**Affiliations:** 1 Internal Medicine, Burjeel Hospital, Abu Dhabi, ARE; 2 Medicine, Burjeel Medical City, Abu Dhabi, ARE; 3 Internal Medicine, Burjeel Medical City, Abu Dhabi, ARE

**Keywords:** acute transverse myelitis (atm), diagnosis of exclusion, inflammatory myelopathy, seronegative autoimmune disease, steroid responsive

## Abstract

Transverse myelitis (TM) is an acquired inflammatory disorder of the spinal cord with a heterogeneous aetiology that includes demyelinating, autoimmune, infectious, granulomatous, and paraneoplastic causes. Although advances in antibody testing, particularly aquaporin-4 (AQP4-IgG) and myelin oligodendrocyte glycoprotein immunoglobulin G (MOG-IgG), have refined the classification of many cases, a significant proportion remain seronegative, presenting diagnostic and therapeutic challenges.

We report the case of a 44-year-old man who presented with progressive fatigue, significant weight loss, lower abdominal numbness, urinary urgency, and intermittent blurred vision. Magnetic resonance imaging of the thoracic spine demonstrated an intramedullary T2 hyperintense lesion at the T10-T11 level with patchy gadolinium enhancement, consistent with inflammatory myelitis. An extensive diagnostic evaluation excluded infectious, systemic autoimmune, granulomatous, endocrine, demyelinating, and paraneoplastic causes. The patient showed marked clinical improvement following high-dose intravenous methylprednisolone, with resolution of sensory and autonomic symptoms and stabilisation of weight, while mild fluctuating fatigue persisted.

This case highlights the diagnostic grey zone of seronegative immune-mediated TM and underscores the importance of systematic exclusion, longitudinal assessment, and therapeutic responsiveness in establishing diagnosis and guiding management.

## Introduction

Transverse myelitis (TM) is an acquired inflammatory myelopathy characterised by acute or subacute motor, sensory, and autonomic dysfunction resulting from focal spinal cord inflammation [1]. The estimated annual incidence ranges from one to eight cases per million population, affecting individuals across a wide age spectrum [1]. The aetiology of TM is heterogeneous and includes multiple sclerosis (MS), neuromyelitis optica spectrum disorder (NMOSD), myelin oligodendrocyte glycoprotein antibody-associated disease (MOGAD), systemic autoimmune diseases, infections, granulomatous disorders, and paraneoplastic syndromes [1].

Over the past decade, the identification of aquaporin-4 immunoglobulin G (AQP4-IgG) and myelin oligodendrocyte glycoprotein immunoglobulin G (MOG-IgG) antibodies has significantly refined the classification of inflammatory myelopathies, allowing many cases previously labelled as idiopathic to be reclassified under defined neuroimmunological entities [2,3]. However, antibody-negative inflammatory myelitis remains within the broader spectrum of immune-mediated myelopathies and continues to pose diagnostic challenges [2,4]. Despite advances in serological testing, approximately 20-30% of patients with inflammatory myelitis remain seronegative for both AQP4-IgG and MOG-IgG and do not fulfil diagnostic criteria for MS or other established demyelinating disorders [5]. These cases occupy a diagnostic grey zone and present challenges in classification, prognostication, and long-term management [2].

Seronegative immune-mediated TM is therefore a diagnosis of exclusion that relies on careful clinical phenotyping, detailed radiological assessment, extensive exclusion of infectious, systemic, neoplastic, and metabolic causes, and close longitudinal observation [2,3]. The clinical course may be monophasic or relapsing, and there are currently no validated biomarkers to reliably predict disease behaviour or relapse risk in this subgroup [6,7].

 We report a case of monophasic, steroid-responsive seronegative immune-mediated TM that illustrates the diagnostic challenges inherent to antibody-negative inflammatory myelopathies. This case is reported to emphasise how systematic exclusion, therapeutic responsiveness, and longitudinal clinical-radiological follow-up together provide diagnostic clarity in the absence of disease-specific biomarkers, a scenario frequently encountered in real-world neuroimmunology practice.

## Case presentation

A 44-year-old previously healthy man presented in August 2025 with a two-month history of progressive fatigue, unintentional weight loss of 6-7 kg, intermittent blurred vision, band-like numbness over the lower abdomen, and urinary urgency. He also reported occasional night sweats but denied fever, cough, rash, joint swelling, or recent travel.

On examination, he appeared tired but hemodynamically stable (blood pressure 130/86 mmHg, heart rate 65 beats per minute, temperature 36.9°C, body mass index 26 kg/m²). The general examination was unremarkable. Neurological examination revealed a sensory level at approximately T10, brisk reflexes in the lower limbs, and subtle imbalance on tandem gait. Motor strength was full (Medical Research Council (MRC) grade 5/5) in all muscle groups. Cranial nerves were intact, including eye movements and facial strength. Visual acuity was near normal with mild subjective blurring; fundoscopy was unremarkable.

Baseline laboratory investigations, including full blood count, renal and liver function tests, electrolytes, glucose, and inflammatory markers, were largely normal or mildly nonspecific.

**Figure 1:**
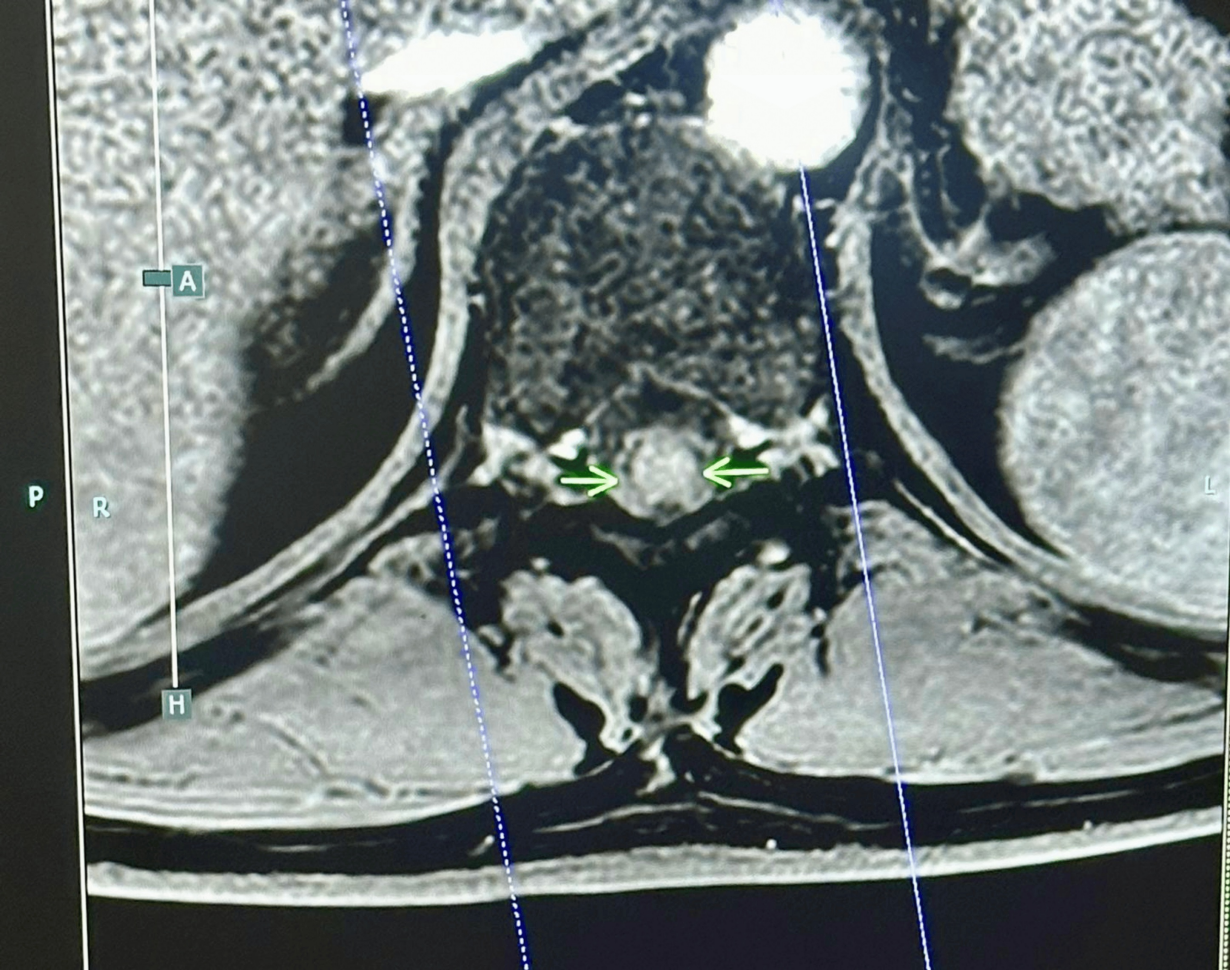
MRI of the spine Focal T2 hyperintense lesion at T10-T11 with patchy gadolinium enhancement involving the thoracic spinal cord, consistent with inflammatory myelitis.

MRI of the spine showed a focal T2 hyperintense lesion at T10-T11 with patchy gadolinium enhancement involving the thoracic spinal cord, consistent with inflammatory myelitis (Figure 1). MRI brain was normal, without white matter plaques, mass lesions, or leptomeningeal enhancement. A working diagnosis of TM was made, and an extensive aetiological workup was initiated.

Investigation and diagnostic journey

Given the combination of constitutional symptoms and a focal spinal cord lesion, the diagnostic workup was broad and systematic, reflecting the extensive differential diagnosis of TM.

Infectious Workup

Because of the patient’s weight loss and night sweats, infectious causes, including tuberculosis and neuroborreliosis, were considered. An initial weakly positive IgM serology for *Borrelia burgdorferi* (IgM 1.2), performed externally, raised suspicion for Lyme disease. However, repeat Lyme ELISA and confirmatory immunoblot testing were negative. Cerebrospinal fluid (CSF) polymerase chain reaction (PCR) for *Borrelia burgdorferi* returned negative results, supporting the absence of active Lyme neuroborreliosis and suggesting that the initial IgM result was likely a false positive.

Tuberculosis was evaluated with an interferon-γ release assay (QuantiFERON-TB Gold), which was negative (Mitogen minus Nil value 9.9 IU/mL, Nil tube 0.1 IU/mL). CT chest showed no pulmonary lesions or mediastinal lymphadenopathy. Viral and other infectious causes were also investigated: serology and/or CSF testing for HSV (HSV-1, 2, and 6 DNA not detected), Varicella-zoster virus (VZV) (VZV DNA not detected), cytomegalovirus (CMV) (with high IgG titres - IgG Antibody of 457 U/mL and CMV IgG of 108 U/mL, as well as high-avidity antibodies of CMV Avidity 76% suggesting past infection rather than acute disease), Epstein-Barr virus (EBV) (IgM and IgG <0.2 AI) and HIV showed no evidence of active infection. C-reactive protein (2.1 mg/L) and erythrocyte sedimentation rate (17 mm/hour) were within normal limits, providing additional evidence against active systemic infection.

Overall, infectious myelitis was considered unlikely on the basis of negative serology, negative CSF PCR, non-inflammatory CSF profile, and unremarkable imaging.

Autoimmune and Systemic Workup

To evaluate for systemic autoimmune or endocrine disease, an extensive panel was conducted. ANA, anti-dsDNA, and extractable nuclear antigen (ENA) panel (including anti-Ro/SSA, anti-La/SSB, anti-Sm, and anti-RNP, which were all <0.2 AI) were negative. Thyroid function tests (TSH 1.74 mIU/L; FT4 17.2 pmol/L), serum cortisol (110 µg/L), ACTH (3.4 pmol/L), and pituitary profile (including prolactin 4.23 ng/mL) were within normal limits. Parathyroid hormone (3.86 pmol/L), vitamin B12 (610 pg/mL), and folate were also normal. Tissue transglutaminase antibodies were negative, excluding coeliac disease, and parietal cell as well as intrinsic factor antibodies (0.8 U/mL) were negative, arguing against autoimmune gastritis. Faecal calprotectin (14 µg/g) was normal, making chronic inflammatory bowel disease unlikely.

There was therefore no evidence of systemic connective tissue disease, vasculitis, or endocrine pathology to explain the myelitis.

Demyelinating Disease Panel

Given the spinal cord involvement, NMOSD and MOGAD were major considerations. AQP4-IgG (NMO antibody) was negative at a titre of 1:10, and MOG-IgG returned negative results at 1:10. Combined with a normal MRI brain and the absence of prior demyelinating episodes, these results made NMOSD and MOGAD unlikely. MS was similarly improbable in the absence of brain lesions, typical demyelinating features, or CSF oligoclonal bands.

Granulomatous Disease

Neurosarcoidosis is a well-known mimic of myelitis and was carefully considered. Serum ACE level (31 U/L) was normal, and serum calcium (2.39 mmol/L) was within the reference range. CT of the chest, abdomen, and pelvis showed no hilar lymphadenopathy, pulmonary infiltrates, or organ involvement suggestive of sarcoidosis. Furthermore, MRI did not show leptomeningeal enhancement or multifocal lesions typical of neurosarcoidosis. These findings did not support a diagnosis of neurosarcoidosis.

Paraneoplastic Evaluation

The triad of weight loss, night sweats, and fluctuating fatigue raised concern for malignancy-associated paraneoplastic myelopathy. An extended paraneoplastic antibody panel was performed and was negative for anti-Hu (ANNA-1), anti-Ri (ANNA-2), anti-Yo (PCA-1), CRMP5/CV2, Ma2/Ta, amphiphysin, ANNA-3 (<1:10), PCA-2 (<1:10), and TR antibodies (<1:10). CT chest, abdomen, and pelvis showed no evidence of mass lesions, lymphadenopathy, or organ involvement. Tumour markers, including CEA (1.1 ng/mL) and CA19-9 (9 U/mL), were within normal limits. Collectively, these findings made paraneoplastic myelitis and occult malignancy highly unlikely.

CSF Analysis

Lumbar puncture demonstrated normal opening pressure, normal cell count and differential, normal protein (0.31 g/dL) and glucose (3.60 mmol/L), and low LDH (13.7 U/L). No oligoclonal bands were detected in CSF or serum, and cytology was negative for malignant cells. The CSF profile was therefore non-inflammatory and nonspecific but did not support infectious, neoplastic, or classical demyelinating disease.

Taken together, the exhaustive infectious, autoimmune, demyelinating, granulomatous, endocrine, and paraneoplastic workup was negative. The absence of systemic disease, in combination with MRI evidence of focal spinal cord inflammation and a non-inflammatory CSF profile, strongly suggested a localised autoimmune process that was seronegative for currently recognised antibodies.

Differential diagnosis 

Over the course of the workup, several conditions were seriously considered and sequentially excluded. Infectious myelitis, including Lyme disease, tuberculosis, and viral causes, was rendered unlikely by negative serology, PCR testing, CSF parameters, and imaging. NMOSD and MOGAD were considered but were not supported by antibody testing, imaging, or clinical evolution. MS was unlikely in the absence of brain lesions, CSF oligoclonal bands, and typical dissemination in time and space. Neurosarcoidosis was not supported by ACE, calcium, or CT imaging. Paraneoplastic myelitis was made unlikely by the negative paraneoplastic antibody panel, unremarkable imaging, and lack of clinical progression. Metabolic and deficiency states, including B12 and folate deficiency, were excluded by normal laboratory values.

Ultimately, the diagnosis of monophasic immune-mediated TM (seronegative subtype) was made based on the clinical picture, MRI findings, exhaustive negative workup, and the pattern of response to therapy (Table 1).

**Table 1:** Summary of diagnostics ACTH, adrenocorticotropic hormone; Ag/Ab, antigen/antibody; CSF, cerebrospinal fluid; CT, computed tomography; DNA, deoxyribonucleic acid; ELISA, enzyme-linked immunosorbent assay; HSV, herpes simplex virus; NMO, neuromyelitis optica; NMOSD, neuromyelitis optica spectrum disorder; T2, transverse relaxation time; T4, thyroxine; TB, tuberculosis; TSH, thyroid-stimulating hormone

Diagnostic Category	Differential Diagnosis	Investigations	Results and Interpretation
Imaging	Transverse myelitis	MRI	Focal T2 hyperintense lesion at T10-T11 with patchy gadolinium enhancement involving the thoracic spinal cord suggesting inflammatory myelitis
Infectious aetiology	Neuroborreliosis	IgM serology for *Borrelia burgdorferi* Lyme ELISA and confirmatory Immunoblot	Initial weakly positive IgM. ELISA and immunoblot Negative
				Tuberculosis	QuantiFERON-TB Gold, CT chest	Negative
	Herpes simplex virus	HSV 1,2 and 6 DNA	Not detected
	Varicella-zoster virus (VZV), cytomegalovirus (CMV)	VZV DNA, CMV DNA	Not detected
	Epstein-Barr virus	IgM, IgG	Negative
	Human immunodeficiency virus	HIV 1,2 Ag/Ab	Negative
	CMV	IgG avidity	High avidity suggesting past infection
	Autoimmune aetiology	Systemic lupus erythematosus, mixed connective tissue disease, Sjögren’s syndrome, systemic autoimmune diseases	ANA, anti-dsDNA, and ENA panel (including anti-Ro/SSA, anti-La/SSB, anti-Sm, and anti-RNP	Negative
Autoimmune thyroid disease (Hashimoto's, Graves'), thyroid myelopathy		TSH, T4	Normal
Adrenal insufficiency (Addison's disease), pituitary-adrenal axis dysfunction		Cortisol and ACTH	Normal
Pituitary or hypothalamic pathology		Prolactin	Normal
Metabolic myelopathy		Parathyroid hormone	Normal
Subacute combined degeneration of the spinal cord		Vitamin B12	Normal
Nutritional or metabolic myelopathy		Folate	Normal
Coeliac disease		Tissue transglutaminase antibodies	Negative
Autoimmune gastritis		Parietal cell and intrinsic factor antibodies	Negative
Inflammatory bowel disease		Faecal calprotectin	Negative
Demyelinating disorder	NMOSD and MOGAD	AQP4-IgG (NMO antibody), MOG-IgG MRI brain	Antibodies negative, MRI Brain normal
Granulomatous disease	Neurosarcoidosis	Serum ACE, calcium, CT chest, CT abdomen, CT pelvis, MRI	
Paraneoplastic evaluation	Paraneoplastic myelitis and occult malignancy	Paraneoplastic panel (anti-Hu, anti-Ri, anti-Yo, CV2, Ma2/Ta, amphiphysin, ANNA-3, PCA-2, TR antibodies), CT chest/abdomen/pelvis, tumour markers (CEA, CA19-9)	All tests are within normal limits. Imaging was normal.
CSF analysis	Non-inflammatory CSF; no demyelination or malignancy	Protein, glucose, cells, oligoclonal bands, cytology	Negative

Treatment

The patient was treated with intravenous methylprednisolone 1 g daily for five days. He demonstrated rapid improvement in lower abdominal numbness and urinary urgency within days of treatment initiation. Weight loss stabilised, appetite improved, and fatigue gradually lessened over subsequent weeks, although mild fluctuations persisted. No new neurological deficits developed during or after therapy.

In view of the favourable clinical response, absence of relapse, and improving functional status, long-term steroid-sparing immunosuppressive therapy was not initiated at that time, with plans for reassessment should relapse occur.

Outcome and follow-up

At approximately six-month follow-up, the patient had made a substantial clinical recovery. Neurological examination was normal, with full strength and intact sensation in all four limbs and no evidence of sphincter dysfunction. Intermittent mild blurred vision persisted but was not associated with objective visual deficits. Residual fatigue was present but significantly improved and did not limit daily activities or occupational function.

No new neurological symptoms developed during follow-up, and there was no clinical evidence of relapse or disease progression. A computed tomography scan of the chest, abdomen, and pelvis performed during follow-up showed no evidence of occult malignancy. The overall course remained monophasic at the time of the last assessment.

## Discussion

This case illustrates the complexity of immune-mediated TM in the absence of identifiable biomarkers and highlights how an extensive but predominantly negative diagnostic workup can nonetheless reveal a genuine immune-mediated inflammatory process. TM represents a heterogeneous clinical syndrome, and despite advances in neuroimmunology, a significant proportion of patients remain seronegative for established antibodies such as AQP4-IgG and MOG-IgG, creating ongoing diagnostic uncertainty [2].

Several observational studies have reported that approximately 20-30% of patients with inflammatory myelitis remain antibody-negative for AQP4-IgG and MOG-IgG and do not fulfil criteria for MS [3-5]. These cases are commonly classified as idiopathic or seronegative autoimmune myelitis and may follow either a monophasic or a relapsing course. Large cohort and centre-based analyses, including both adult and paediatric populations, have demonstrated that a substantial subset of TM cases do not evolve into defined demyelinating diseases and may instead represent isolated immune-mediated events with favourable long-term outcomes [5,6].

Flanagan and colleagues have proposed the concept of “autoimmune myelopathies” as a broad spectrum encompassing NMOSD, MOG antibody-associated disease (MOGAD), and seronegative autoimmune myelitis, noting that seronegative cases generally demonstrate better functional outcomes and lower relapse rates than AQP4-IgG-positive NMOSD [2,3]. Similarly, comparative studies examining NMOSD and MOGAD have shown that antibody-positive disorders are more likely to follow a relapsing course, whereas seronegative presentations may remain monophasic, particularly in the absence of characteristic brain or optic nerve involvement [7]. The benign clinical course and favourable neurological recovery observed in this patient are consistent with these observations.

It is important to acknowledge the inherent limitations in classifying inflammatory myelitis in the absence of disease-specific biomarkers. While the lack of positive immunological, CSF, or pathognomonic radiological markers precludes definitive autoimmune attribution, current clinical practice increasingly recognises a subset of immune-mediated myelopathies that remain seronegative despite comprehensive testing. In such cases, diagnosis relies on systematic exclusion of alternative aetiologies, inflammatory imaging features, therapeutic responsiveness, and longitudinal clinical stability rather than on a single confirmatory test. The present case is therefore best understood within this diagnostic framework, highlighting the practical reality that immune-mediated central nervous system inflammation may exist beyond currently detectable antibody profiles.

Paraneoplastic myelitis was an important consideration in this case, given the presence of constitutional symptoms including weight loss, night sweats, and fluctuating fatigue. Paraneoplastic myelopathies typically present with subacute, progressive neurological decline, are often associated with longitudinally extensive spinal cord lesions, and frequently show limited or transient responses to corticosteroid therapy. They are most commonly associated with small-cell lung cancer, thymoma, breast cancer, or testicular tumours and are often accompanied by detectable onconeural antibodies [8]. In this patient, the absence of paraneoplastic antibodies, normal cross-sectional imaging, lack of progressive neurological deterioration, and sustained clinical improvement following high-dose corticosteroids argue strongly against a paraneoplastic aetiology.

Similarly, neurosarcoidosis is a well-recognised mimic of inflammatory myelitis and warrants careful exclusion. It typically manifests with systemic involvement, pulmonary disease, and characteristic neuroimaging features such as leptomeningeal enhancement or multifocal intraparenchymal lesions. In this case, normal serum angiotensin-converting enzyme levels, normal calcium levels, absence of thoracic or abdominal lymphadenopathy on imaging, and lack of characteristic magnetic resonance imaging features did not support a diagnosis of neurosarcoidosis [9].

The patient’s benign course and good recovery are consistent with reports that seronegative immune-mediated myelitis often carries a more favourable prognosis compared with NMOSD and some forms of MOGAD, which are more frequently associated with relapses and residual disability [10]. In particular, MOG antibody-associated disease may demonstrate distinct imaging patterns and relapse tendencies that were not observed in this patient, further supporting the classification of this presentation as monophasic seronegative autoimmune TM.

This case also underscores several important diagnostic principles relevant to atypical inflammatory demyelinating syndromes of the central nervous system. First, negative serological testing does not exclude autoimmune disease. The absence of AQP4-IgG and MOG-IgG antibodies does not preclude an immune-mediated inflammatory process, particularly when clinical presentation, imaging findings, and treatment response are highly suggestive. As understanding of neuroimmunology continues to evolve, it is increasingly recognised that not all pathogenic antibodies have been identified or are detectable using currently available assays [11].

Second, weakly positive infectious serologies must be interpreted with caution and in the context of the overall clinical picture. In this case, an initial low-titre Lyme immunoglobulin M result raised concern but was not corroborated by confirmatory testing, CSF analysis, or compatible imaging findings. Overreliance on isolated serological results could have led to misdiagnosis and inappropriate antimicrobial therapy.

Third, steroid responsiveness can be both therapeutically beneficial and diagnostically informative. The rapid and sustained improvement observed following high-dose intravenous methylprednisolone strongly supported an inflammatory, immune-mediated aetiology rather than degenerative, vascular, or neoplastic processes, which typically do not respond so dramatically to corticosteroids.

Finally, time itself proved to be an essential diagnostic tool. The absence of relapse, radiological resolution of the spinal cord lesion, and lack of emergence of systemic disease over follow-up provided reassurance and helped exclude occult malignancy and evolving demyelinating disease. This longitudinal stability strongly favoured a diagnosis of monophasic seronegative immune-mediated TM.

From a pathophysiological standpoint, seronegative autoimmune myelitis may represent a localised, antibody-undetected autoimmune process, possibly driven by T-cell-mediated mechanisms or by antibodies not routinely captured by standard assays. The clinical importance of recognising this entity lies in its potential for good recovery with prompt immunotherapy and careful follow-up, rather than defaulting to prolonged empirical treatments or attributing symptoms to functional or non-organic causes once conventional antibody tests are negative.

Clinical implications include recognition that it is appropriate to accept a diagnosis of seronegative autoimmune TM once a comprehensive evaluation has excluded other major entities such as infection, malignancy, MS, NMOSD, MOGAD, and neurosarcoidosis. Not all patients with TM require immediate long-term immunosuppression; in monophasic, improving patients, careful observation with magnetic resonance imaging surveillance may be sufficient. Persistent fatigue is a common sequela of TM and should be addressed with supportive measures and realistic counselling, rather than being automatically interpreted as ongoing active disease in isolation.

Future research directions include the identification of novel antibodies and immune targets in seronegative myelitis through advanced serological techniques and tissue-based assays, the development of biomarkers to distinguish monophasic from relapsing seronegative myelitis, and prospective studies in under-represented regions such as the Middle East to better understand epidemiology and phenotype in different ethnic populations. Mechanistic studies on post-inflammatory fatigue in TM survivors would also improve the holistic management of these patients.

## Conclusions

This case demonstrates that seronegative immune-mediated TM can be diagnosed through systematic exclusion, characteristic imaging findings, steroid responsiveness, and careful longitudinal follow-up, even in the absence of disease-specific antibodies. A negative diagnostic workup does not exclude an inflammatory aetiology when clinical evolution and treatment response are concordant. This case highlights the importance of structured evaluation, cautious interpretation of negative investigations, and follow-up over time to confirm a monophasic course and avoid unnecessary long-term immunosuppression.
